# Exploration forays in juvenile European hares (*Lepus europaeus*): dispersal preludes or hunting-induced troubles?

**DOI:** 10.1186/1472-6785-14-6

**Published:** 2014-02-26

**Authors:** Alexis Avril, Jérôme Letty, Yves Léonard, Dominique Pontier

**Affiliations:** 1 Office National de la Chasse et de la Faune Sauvage (ONCFS), Direction des Études et de la Recherche, F-34990 Juvignac, France; 2 ONCFS, F-45370 Dry, France; 3 Laboratoire de Biométrie et Biologie évolutive, CNRS UMR5558, Université de Lyon Université Claude Bernard Lyon 1, F-69622 Villeurbanne, France; 4 Present address: Centre for Ecology and Evolution in Microbial Model Systems, Linnaeus University, Kalmar SE-391 82, Sweden

**Keywords:** Predation risk, Extra home-range movements, Telemetry, Dispersal stage, Lagomorphs

## Abstract

**Background:**

Movements of animals have important consequences, at both the individual and population levels. Due to its important implications in the evolutionary dynamics of populations, dispersal is one of the most studied types of movement. In contrast, non-permanent extra home-range movements are often paid less attention. However, these movements may occur in response to important biological processes such as mating or predation avoidance. In addition, these forays are often preludes to permanent dispersal, because they may help individuals gain cues about their surroundings prior to settlement in a new place.

In the European hare, exploration forays occur predominantly in juveniles, the time at which most hares disperse. In France, the timing of dispersal also overlaps with the hare hunting period. However, the determinants of such behaviour have not yet been studied. Herein, we investigate whether these non-permanent explorations are dispersal attempts/preludes or, in contrast, whether they are triggered by other factors such as disturbances related to hunting.

**Results:**

Contrary to natal dispersal, we did not find strong male-bias in the propensity to engage in explorations. Exploration forays occurred less in juveniles than in adults and later in the season than natal dispersal. This was the case both for philopatric movements and for movements occurring after dispersal and settlement. These movements were also more likely to occur during the hare hunting period and the mating season.

**Conclusions:**

We suggest that explorations in hares are triggered by factors other than dispersal and that hares may respond to hunting disturbances. Overall, we emphasize the need to account for human-related predation risk as a factor driving space-use in harvested species.

## Background

Movements of animals underly many biological processes from the individual level (e.g., home ranging, mating, foraging) to the population level (e.g., population persistence, population connectivity, invasion, disease spread) [1]. Because of its strong implication in the evolutionary dynamics of populations, one of the most studied types of movement in ecology is dispersal, i.e. the permanent movement of juveniles from their birth place to the place of their first breeding attempt (natal dispersal) or the movement of adults between two breeding places (breeding dispersal) [2,3]. Another well described extra home-range movement concerns non-permanent forays. These movements are often related to foraging and/or mating activities [4,5], but also temporary escape into refuges due to increasing disturbance in the surroundings [6].

Exploration of surroundings is a key determinant step in realized dispersal. Dispersal consists of three inter-dependent stages: departure from the place of origin, exploration (the so-called transient stage) and settlement into a new place [7]. Dispersal across and into unfamiliar habitat may be costly owing to high energy expenditure, exposure to predators and ignorance of future settlement habitat quality [8,9]. The cost/benefit ratio of dispersal depends mostly on the suitability of the settlement place relative to the place of origin [10]. Exploration may help the animal gain cues about their surroundings [11,12], this in turn buffers the potential costs of dispersal. The conditions encountered during this exploratory stage are likely determinants in the animal’s decision to settle in a given place, pursue the transient-exploration stage, or abort dispersal. For instance, unavailable vacant places or sexual partners in the range of dispersal distances, frequent aggressive encounters with residents in high density patches (the so-called ‘social fence’ hypothesis [13]) or increasing disturbance (e.g. predation pressure [14,15]) during the exploration stage may reduce the chance of permanent dispersal and compel the individual to remain philopatric. Identifying the factors that may affect the success or failure of settlement is crucial to our understanding of dispersal efficiency in a given species. In particular, in the context of harvested populations, evaluating the influence of recreational hunting activities on species dispersal is of prime importance, for both conservation and management.

The European hare, *Lepus europaeus*, is a declining game mammal encountered in farmland habitats. Previous work has shown that hunting has direct effects on hares’ dispersal. Dispersal in European hares occurs predominantly in juveniles, from the end of summer until the end of autumn, overlapping with the beginning of hare hunting (autumn) [16,17]. The close overlap between dispersal and hunting periods has been shown to reduce survival of dispersing hares relative to philopatric individuals, with dispersers suffering a higher risk of being shot during the transient stage [18], in addition to the risk of being killed by natural predators (mainly red fox, *Vulpes vulpes*) [19]. In contrast, non-lethal and indirect effects of hunting on hare movement and dispersal are still poorly documented and controversial. Temporary forays outside the usual home-range have been observed in hares. In juveniles, most forays have been recorded during autumn, at the beginning of the hare hunting season [18,20], and may or may not followed by permanent dispersal. Given that these extra home-range movements were recorded together with dispersal events, we might expect them to be movements performed by potential dispersers searching for settlement places, but failing to find suitable ones and returning back to their site of origin. Alternatively, since these forays were most often recorded during the hunting period, they could be triggered by hunting disturbances. For example, they could be searches for, or escapes into refuges, and thus triggered by different endogeneous and exogeneous factors than those triggering juvenile dispersal.

We conducted a three-year radio-tracking study of European hares in a high density hunted population, to investigate whether (1) explorations in juvenile hares were dispersal preludes or failed dispersal attempts, or, in contrast, (2) whether these movements were mainly triggered by increasing disturbances related to hunting. Regarding the first prediction, movements of dispersers and explorers should share similar triggering factors: if explorations are dispersal attempts or preludes to dispersal, we would expect the date of departure for explorations to overlap with that of realized dispersal events, and for exploration rates to be biased toward males given that natal dispersal rates are male-biased. By considering the period when most explorations occur, we were able to gain insights into the plausible role of hunting in settlement failure. Under the second scenario, i.e. if explorations are potentially triggered by hunting disturbances, we would expect more explorations to occur during the hunting period than during the rest of the year, regardless of the age or sex of individuals, and the propensity of undertaking explorations would be similar between philopatric individuals (i.e. those that have never left their birth place) and individuals that have already dispersed and settled in a new home-range.

## Methods

### Species and study site

We studied the movements of European hares using radio-telemetry between 2003 and 2005, in a hunted, high density population (41 hares/km^2^) in France. The study site was located in an intensive cropping area in the region Centre, near Blois (France, 47°44′35″ N, 1° 21′ 55″ E). In this population, the hare hunting period begins each year on the last weekend of September (close to the 267th Julian day of the year) and lasts until the end of the year.

The European hare is a medium sized mammal, living in temporary feeding groups with no stable social structure, and the mating system is polygynous-promiscuous [21]. As in other polygynous-promisucous species, natal dispersal rates are male-biased [16,17]. In our population, male dispersal rates were on average twice as high as female dispersal rates, and most dispersal events occured in juveniles as they reached adult size [17] i.e. between 3 and 5 months old [22,23]. The mating season generally starts in midwinter (January-February) and lasts until midsummer, occasionally until September [23,24].

### Radio-telemetry, movement types and individual selection

Two hundred and fifteen hares (184 juveniles and 31 adults) were trapped using unbaited boxes [25] during monthly trapping sessions from spring to the end of summer each year (see Additional file 1). The traps were checked on a daily basis, and once a hare was caught, it was sexed, weighed and fitted with an eartag (Presadom; Chevillot, Albi, France) and a radiocollar (TW-5 Biotrack, Wareham, UK and TXH-2, Televilt, Lindsberg, Sweden; 50 g, 1500 m range, battery life 16 months) and then released. Radio-locations were recorded by triangulation, usually once a week and always during the day when most hares rest in their den sites [26]. Age at first capture was assessed from body mass for young individuals (juveniles <180 days old, yearlings >180 days old but born during the year of capture) and length of hind foot for adults (> 1 year old) (see [16,17,27] for further details).

In our population, we defined individual’s movements remaining within a 588 m radius from the centre of the estimated home-range of origin as philopatric movements [17]. We chose a 588 m threshold distance because it was the usual home-range radius estimated using the locations of adults monitored in our population during the three years study (details about birth site estimates, and home-ranges are given in [17,18]).

**Figure 1:**
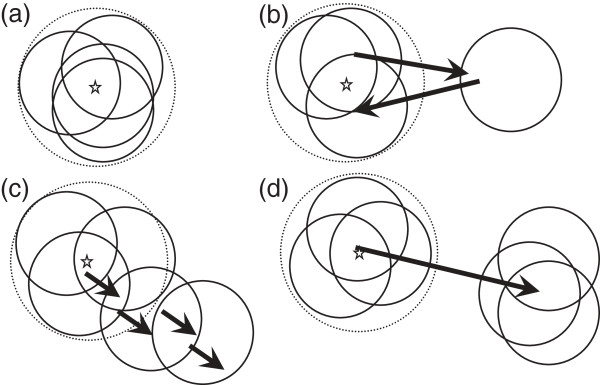
**Typical types of space use in the European hare *Lepus europaeus *(following McShea & Madison [**[28]**]).** Dashed circle: 588 m circle radius defining the home-range of origin; the centre of the home-range of origin (star) is the arithmetic centre of the first locations within the 588 m circle radius. **(a)** ‘Stationary’: each home-range (plain circles) overlaps previous estimates; **(b)** ‘Explorer’: quasi ‘Stationary but the hare makes short-time extra home-range forays with returns later on; **(c)** ‘Shifter’ disperser: the individual gradually leaves its home-ranges of origin; **(d)** ‘One-way’ disperser: the individual suddenly changes its home-range by making a one-way and permanent movement and settling in new disjunctive home-range.

Following a classification of the different types of space use in small mammals [28], we identified four kinds of movement types in hares: (1) ‘Stationary’ (Figure 1a), whereby the individual remained inside the home-range of origin (i.e. within the 588 m radius around the centre of the home-range of origin); (2) ‘Explorer’ (Figure 1b), whereby the individual made one or more temporary forays outside their home-range of origin (i.e. beyond the 588 m radius from the centre of the home-range of origin); (3) ‘Shifter’ (Figure 1c), whereby the individual left progressively its home-range of origin (and moved permanently beyond the 588 m radius from the centre of the home-range of origin) and (4) ‘One-way’ (Figure 1d), whereby the individual undertook a brief (< 7 days) and permanent departure from the home-range of origin and settled in a new disjunctive area (at least 588 m beyond the centre of the home-range of origin). ‘Stationary’ and ‘Explorer’ types correspond to philopatric individuals, whereas ‘Shifter’ and ‘One-way’ describe dispersing individuals, i.e. those that permanently departured from the home-range of origin.

To analyse movement type, we considered adults and juveniles that were monitored for at least three months and 10 locations. For juveniles, we only considered individuals (i) <90 days old at the time of their first capture, to exclude immigrants (70% of dispersal departures occur between the ages of 120 and 180 days) and (ii) monitored until at least 150 days old, at which time they have reached adult body weight [23]. These conditions were necessary to avoid ambiguity in movement type interpretation and to confidently assess the home-range of origin for each individual. We also excluded all individuals showing significant departure following their capture, so as to not confound natural dispersal from possible trapping-induced dispersal. At the end of these steps, the dataset was composed of 87 juveniles (n = 26 ‘Stationary’, 31 ‘Explorer’, 23 ‘One-way’, 7 ‘Shifter’) and 12 adults (5 ‘Stationary’, 5 ‘Explorer’ and 2 ‘One-way’).

**Figure 2:**
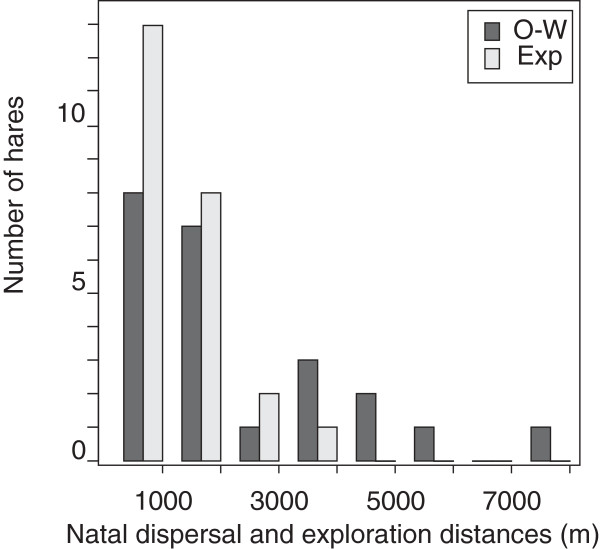
**Distance moved by juveniles from home-range of origin (*Dm*) centre during ‘one-way’ dispersal movements (‘O-W’. dark gray bars) and first exploration movements at more than 704 m from the centre of the home-range of origin (‘Exp’. light gray bars).**

In the present study, which aims to test whether temporary forays could be dispersal preludes, we considered as explorations only movements performed by ‘Explorer’ individuals > 704 m from the centre of the home-range of origin, i.e. at a distance at least equal to the minimum realized dispersal distance observed in our population [17] (Figure 2). Such a rule prevented us from assigning forays performed close to the periphery of the home-range of origin (i.e. ~ 588 m) as dispersal events, rather than random extra home-range movements.

### Statistical analyses

#### ***Date of departure in juvenile dispersers and explorers***

We investigated the effect of the movement type (‘One-way’ and ‘Explorer’) on the date of departure *Dd* (in days since the beginning of the year) using the 22 ‘Explorers’ making forays > 704 m and the 23 “One-way” dispersers identified in juvenile hares [17]. For ‘One-way’ dispersal types, *Dd* was given by the date of dispersal (number of days since the beginning of the birth year), i.e. the date when the individual was seen for the last time within its home-range before dispersing permanently. For ‘Explorer’ types, we considered the first exploration made by the animal as the timing of the dispersal prelude: we used the date when the individual was seen for the last time before making the first foray beyond the 588 m radius from the centre of the home-range of origin. In both types, *Dd* was given by the date when the individual temporarily or permanently left its usual home-range and moved at least > 704 m from the centre of its home-range of origin. Futhermore, we did not consider explorations prior to dispersal “One way”, as only one individual was observed making this prior dispersal movement.

Juvenile hares were captured in different cohorts in the year, from May to September (‘Explorer’ females (n = 16), Julian date range = [125–264]; ‘Explorer’ males (n = 8), range = [128–257], ‘One-way’ females (n = 7), range = [168–238]; ‘One-way’ males (n = 16), range = [114–250]). Since dispersal of hares is age-related, *Dd* is strongly related to the age of hares at the time of their capture. Age of hares at the time of capture (estimated from body mass) was not precise enough to provide a reliable estimate of age in days. Therefore, we investigated the effect of the movement type *movtyp* and *sex* on *Dd* after accounting for body mass at the time of capture (*BM)* and the date of capture (*Dc)* using an ANCOVA model log(Dd) = BM + sex + Dc*movtyp. In this model, the relationship between *Dd* and *Dc* is used as a surrogate for age-effects after having controlled for potential biases such as trends in (i) *BM* according to *Dc* (suggesting varying ages according to *Dc*), or (ii) toward one particular movement type (see Additional file 2).

The response variable was log-transformed before analysis because the distribution of *Dd* was skewed toward the right. We did not test for higher interaction terms because of the small sample size. We included the interaction Dc*movtyp to specifically investigate the dispersal attempt/prelude hypothesis. Indeed, if both movement types are triggered by similar factors (i.e. under the hypothesis that explorations are dispersal attempts/preludes) we expect no difference of *Dd* regardless of the type after accounting for *Dc* and *BM* (dispersal attempt), or *Dd* varying with *Dc* and *movtyp* in an additive way, exploration departure occurring earlier than recorded ‘One-way’ departures (dispersal preludes). In contrast, if explorations were not related to dispersal, but instead triggered by hunting, we expected the interaction Dc*movtyp to be significant, with explorations occurring preferentially during the hunting season. We selected the factors explaining variation in *Dd* using the Akaike Information Criterion corrected for small sample sizes (AICc) [29]. Model selection was divided in two steps. In step 1, starting from the general model above, we investigated whether adding *BM* improved the fit of the model*.* Then, in step 2, starting from the models retained in step 1, we selected the best model among variants including the factors *Dc, sex* and *movtyp*. We used R software v.2.12 for all analyses [30].

#### ***Factors affecting the probability of making explorations in philopatric individuals and dispersers after settlement***

We explored the influence of *sex*, *age,* period (*per*) and the dispersal state (*disp*) on the probability of a hare temporarily moving more than 704 m from the centre of its usual home-range (noted *dexp*) using logistic models. We ran the analysis on a dataset composed of philopatric individuals (i.e. ‘Stationary’ and ‘Explorer’; n = 57 juveniles, n = 10 adults) and dispersing juvenile (i.e. ‘One-way’; n = 22) after removal of individuals for whom their new home-range could not be clearly identified. These two groups of individuals constituted the two factor level *disp.* The age of hares was modelled as a three modalities factor: juveniles < 180 days old; yearlings < 1 year old, marked as juveniles and monitored during adult stages, and adults marked as adults (> 1 year old). The three age levels helped to disentangle yearlings experiencing their first hunting season and prime-age adult hares having already experienced hunting in the past. We divided the year into three different periods. The pre-hunting period, April-September, relates to crop harvesting and the end of the hare mating season. The second period is the hare-hunting period and lasts from October to December. Finally, January-March is the post-hunting period, characterized by hare mating activity and roe deer drive hunts.

In this analysis, the response variable was binary and coded for each hare, the presence (1) or absence (0) of at least one exploration departure for a given combination of factor levels. Because there could be up to five observations per hare, we accounted for repeated observations using generalized linear mixed models (GLMM). We specifically tested two competitive hypotheses, using two sets of models. The first set specifically tested for the dispersal attempt/prelude hypothesis (hypothesis 1) by considering the influence of *sex*, *age* and *disp* on *dexp*. If explorations are dispersal preludes, we expect the probability of undertaking an exploration to be male-biased, juvenile specific, and occurring preferentially in philopatric individuals. The second set of models investigated the influence of the period on *dexp* (hypothesis 2) starting from the best models in step 1. If explorations were triggered by hunting for instance, we expect the probability of undertaking an exploration to be higher during the hunting period and to involve both philopatric and dispersers after settlement.

In each step, we selected for the best model among the set of candidate models using AICc. Because AIC-based model selection requires maximum likelihood estimates [29], GLMM were fitted using the Gauss-Hermite approximation and the lme4 package for R software 2.12 [30], which gives the fitted model likelihood. We did not test for high interaction terms because of the sparseness of the data and because high interactions led to complete separation of the data and convergence problems. For instance, we could not test for the interaction age*per or age*disp because the levels of the factors were highly correlated and/or led to perfect but probably falsely fit [31]. Then, we started with the model logit(dexp) = age*sex + sex*disp and we resorted on deviance bootstraps after refitting the best models 1,000 times to test for lack of fit [31]. If several models equally fitted the data, we evaluated the relative variable importance and computed the model averaged predictions and their 95% confidence intervals using the R package MuMIn.

#### ***Effect of period on distances moved***

We also investigated the influence of period through the use of a continuous response variable and a linear model. We used distances from the centre of the home-range of origin (*Dm*) as the measure of individual exploration, both in philopatric and dispersers after settlement. Distances were transformed using the Box-Cox transformation (λ = 0.17, Kolmogorov Smirnov test based on 10,000 sample size, p = 0.08), because they were highly skewed. We accounted for consecutive repeated measures of *Dm* within a given individual using a linear mixed model with an AR1 correlation structure (ρ = 0.43). We selected the main factors that explained variation in the distances in a two step approach. In the first step we selected for the best model structure among variants including the factors *sex*, *age* (juveniles, yearling and adults) and dispersal state (*disp*) (hypothesis 1). We started with the model Dm = age*sex + sex*disp to account for the fact that juvenile males are more prone to move greater distances in the dispersal group, since natal dispersal is male biased. Then, starting from the best model selected in the previous step, we specifically investigated the influence of hunting by adding the factor *period* (hypothesis 2). The interaction age*disp was not tested because of convergence problems (no settled dispersers in adult stage). Model selection was performed using AICc. When several models equally fitted the data, we evaluated the relative variable importance and computed the model averaged predictions as described above.

## Results

### Effect of movement type on the date of departure

Including the explanatory variable body mass at the time of capture (*BM*) from the starting model log(*Dd*) = BM + sex + Dc*movtyp, did not improve the model fit (ΔAICc = 1.84, Table 1, step 1). Hence, we investigated the influences of *Dc, sex* and *movtyp* on variations in the date of departure *Dd*, starting from these two previous models. In step 2, the models log(*Dd*) = sex + Dc*movtyp and log(*Dd*) = BM + sex + Dc*movtyp were still the best models among all possible variants (Table 1, step 2). The simpler model showed a significant interaction Dc*movtyp (F = 6.26, d.f. = 1, p = 0.02) and a significant additive effect of *sex* (F = 4.70, d.f. = 1, p = 0.04). As expected, there was a positive and significant relationship between *Dd* and *Dc* in both types, but the slope was higher for ‘One-way’ departures than for ‘Explorer’ (‘Explorer’ slope: 1.5*10e-03 ± 0.7*10e-03 ( ± SE), student t = 2.28, d.f. = 42, p = 0.028; ‘One-way’ slope: 4.2*10e-03 ± 1.3*10e-03 (± SE), student t = 3.31, d.f. = 42, p < 10e-03).

**Table 1:** **Models tested for the date of departure ( *Dd *) according to *sex*, the movement type ( *movtyp *) (One-way dispersers or philopatric explorer), the date of capture ( *Dc *) and body mass ( *BM *), based on AICc and Akaike weights (*w***_***i***_**)**

***Models***	***np***	***Deviance***	***AICc***	***ΔAICc***	***w***_***i***_
**Step1**					
log(Dd) = sex + Dc* movtyp	**6**	**-51.82**	**-37.61**	**0**	**0.72**
log(Dd) = BM + sex + Dc* movtyp	**7**	**-52.8**	**-35.77**	**1.84**	**0.28**
**Step2**					
Log(Dd) = sex + Dc*movtyp	**6**	**-51.82**	**-37.61**	**0**	**0.4**
Log(Dd) = BM + sex + Dc*movtyp	**7**	**-52.8**	**-35.77**	**1.84**	**0.16**
Log(Dd) = BM + Dc*movtyp	6	-50.06	-35.86	1.76	0.16
Log(Dd) = Dc*movtyp	5	-46.82	-35.29	2.33	0.12
*Log(Dd) = Dc + Sex + movtyp*	5	-45.76	-34.22	3.39	0.07
*Log(Dd) = BM + Dc + Sex + movtyp*	6	-46.56	-32.34	5.27	0.03
*Log(Dd) = BM + Dc + movtyp*	5	-43.78	-32.23	5.38	0.03
*Log(Dd) = Dc + movtyp*	4	-40.86	-31.86	5.75	0.02
*Log(Dd) = Dc + sex*	4	-38.56	-29.55	8.06	0.01
*Log(Dd) = BM + Dc + sex*	5	-38.56	-27.03	10.58	0
*Log(Dd) = Dc*	3	-28.68	-22.1	15.51	0
*Log(Dd) = BM + Dc*	4	-30.1	-21.09	16.52	0
*Log(Dd) = BM*	3	-16.88	-10.3	27.31	0

Models in bold character are those having the best support from the data in each step.

**Figure 3:**
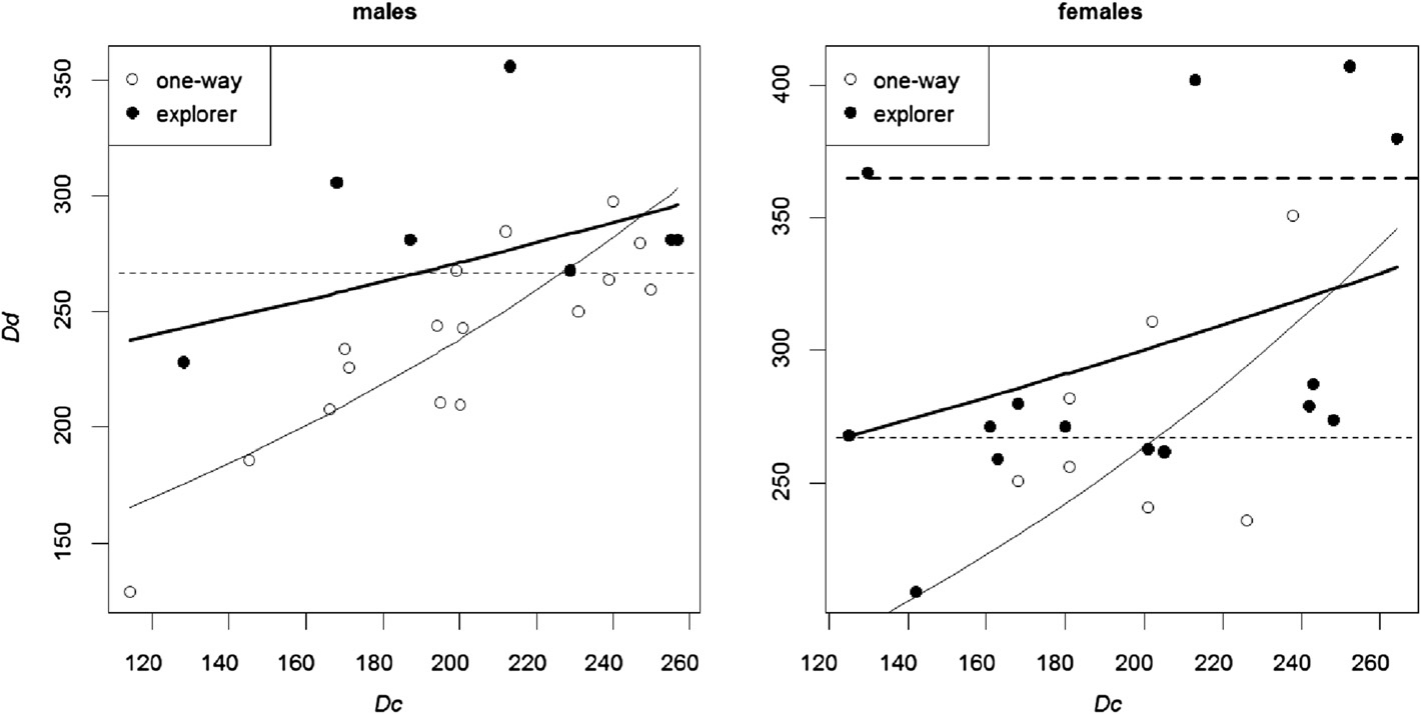
**Date of departure (*Dd*) for juvenile ‘One-way’ dispersers (open circles) and date of first foray departure for juvenile ‘Explorer’ (full circles) according to sex and date of capture (*Dc*).** The date is the number of days since the beginning of the birth year for each hare. Solid lines are back-transformed predictions for ‘One-way’ departures (thin lines) and for ‘Explorer’ departures (thick line). Dashed lines show the beginning (thin) and the end (bold) of the hare hunting period.

Overall, exploration departures were recorded on average one month later than dispersal events (*Dd* ranging from days 209 to 407 for explorations, from 129 to 351 for ‘One-way’), ‘One-way’ departures occurred earlier in males than in females (*Dd* ranging from 129 to 298 vs. from 236 to 351; respectively), whereas the between-sex difference was weak in ‘Explorer’ (*Dd* ranging from 228 to 356 vs. 209 to 407 for males and females respectively (Figure 3)). For the mean *Dc* (201), the mean *Dd* estimates given by the best model were 239, 95% CI [225–256] for ‘One-way’ males, 262 95% CI [243–288] for ‘One-way’ females, 274, 95% CI [253–298] for ‘Explorer’ males and 300, 95% CI [283–321] for ‘Explorer’ females.

### Effect of period on exploration movements

**Figure 4:**
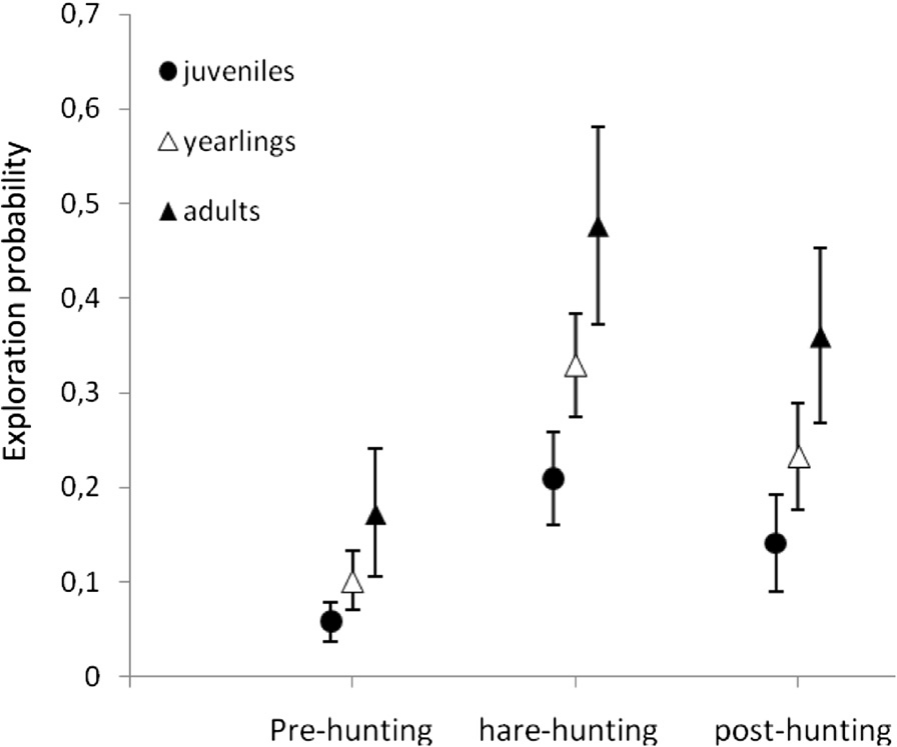
Probability for a hare to make exploration (±SE) according to the age class and the period of the year: pre-hunting: end of hare breeding season and crop harvesting period (March-September); hare-hunting: hare hunting season (October-December); post-hunting: hare mating season and roe deer drive hunts (January-April).

Among the whole set of models assessing the effect of *sex*, *age* and dispersal status on variation in exploration rates (hypothesis 1, Table 2), the models *logit(dexp) = age*, *logit(dexp) = age + sex* and *logit(dexp) = age + disp*, were retained as the most plausible, as their AICc weights summed to 75% and they did not differ by more than 2 AICc points from the higher ranked model. We therefore used these three best models to specifically investigate the influence of period on exploration probability (hypothesis 2, Table 2). Adding the factor *period* always improved the fit of the three previous starting models (ΔAICc > 10 with respect to models in the first step). Among the whole set of models testing for the influence of the period, the models logit(dexp) = age + per, logit(dexp) = age + per*disp, logit(dexp) = age + sex + per,logit(dexp) = age + disp + per and logit(dexp) = per, were retained as the most plausible since they differed by less than 2 AICc points from the best supported model *logit(dexp) = age + per*. The model *logit(dexp) = age + per* provided similar parameter estimates when compared to model averaging (Additional file 3). Estimates given by that model showed that exploration rate was highest during the hare hunting period regardless of the age of hares (e.g. in juveniles: 0.05 ± 0.02; 0.21 ± 0.05; 0.14 ± 0.05 during pre-hunting, hare-hunting and post-hunting periods respectively; estimates ± SE), and that both yearlings and adults have on average higher exploration rates than juveniles (yearlings: 0.10 ± 0.03; 0.33 ± 0.05; 0.23 ± 0.06, adults: 0.17 ± 0.07; 0.48 ± 0.10; 0.36 ± 0.09; during pre-hunting, hare-hunting and post-hunting periods respectively) (Figure 4). Further, using estimates given by the second highest ranked model, we found a significant interaction between the factors *age* and *disp.,* dispersers after settlement having lower exploration rates than philopatric individuals during the post-hunting season (e.g. in yearlings: 0.07 ± 0.06 vs. 0.32 ± 0.08). The sex-effect observed in the set of the most plausible models always described a non-significant male bias in the propensity for undertaking explorations (e.g. *logit(dexp) = age + sex + per,* male intercept difference from female: 0.26 ± 0.37, student t = 0.71, d.f. = 1, p = 0.47).

**Table 2:** **Model selection for the probability for making explorations ( *dexp *) based on AICc and Akaike weights (*w***_***i***_**)**

***Models***	***np***	***Deviance***	***AICc***	***ΔAICc***	***w***_***i***_
**Hypothesis 1**					
***logit(dexp) = age***	**4**	**310.25**	**318.4**	**0.00**	**0.38**
***logit(dexp) = age + sex***	**5**	**309.36**	**319.6**	**1.18**	**0.21**
***logit(dexp) = age + disp***	**5**	**310.11**	**320.3**	**1.94**	**0.15**
*logit(dexp) = (.)*	2	317.77	321.8	3.43	0.07
*logit(dexp) = sex*	3	316.46	322.5	4.16	0.05
logit(dexp) = age + sex*disp	7	308.65	323.0	4.65	0.04
logit(dexp) = age*sex	7	308.78	323.2	4.78	0.03
*logit(dexp) = disp*	3	317.74	323.8	5.44	0.03
*logit(dexp) = sex + disp*	4	316.25	324.4	6.00	0.02
logit(dexp) = age*sex + disp	8	308.35	324.8	6.46	0.02
logit(dexp) = sex*disp	5	316.15	326.4	7.97	0.01
logit(dexp) = age*sex + sex*disp	11	308.15	326.8	8.39	0.00
**Hypothesis 2**					
***logit(dexp) = age + per***	**6**	**294.25**	**306.5**	**0.00**	**0.33**
logit(dexp) = age + per*disp	**9**	**288.77**	**307.4**	**0.85**	**0.21**
***logit(dexp) = age + sex + per***	**7**	**293.52**	**307.9**	**1.37**	**0.17**
***logit(dexp) = age + disp + per***	**7**	**293.84**	**308.2**	**1.69**	**0.14**
***logit(dexp) = per***	**4**	**300.36**	**308.5**	**1.96**	**0.12**
logit(dexp) = age + sex*per	9	293.00	311.6	5.08	0.03
*logit(dexp) = age*	4	310.25	318.4	11.84	0.00
*logit(dexp) = age + sex*	5	309.36	319.6	13.02	0.00
*logit(dexp) = age + disp*	5	310.11	320.3	13.78	0.00

Models in bold character are those having the best support from the data in each hypothesis.

**Figure 5:**
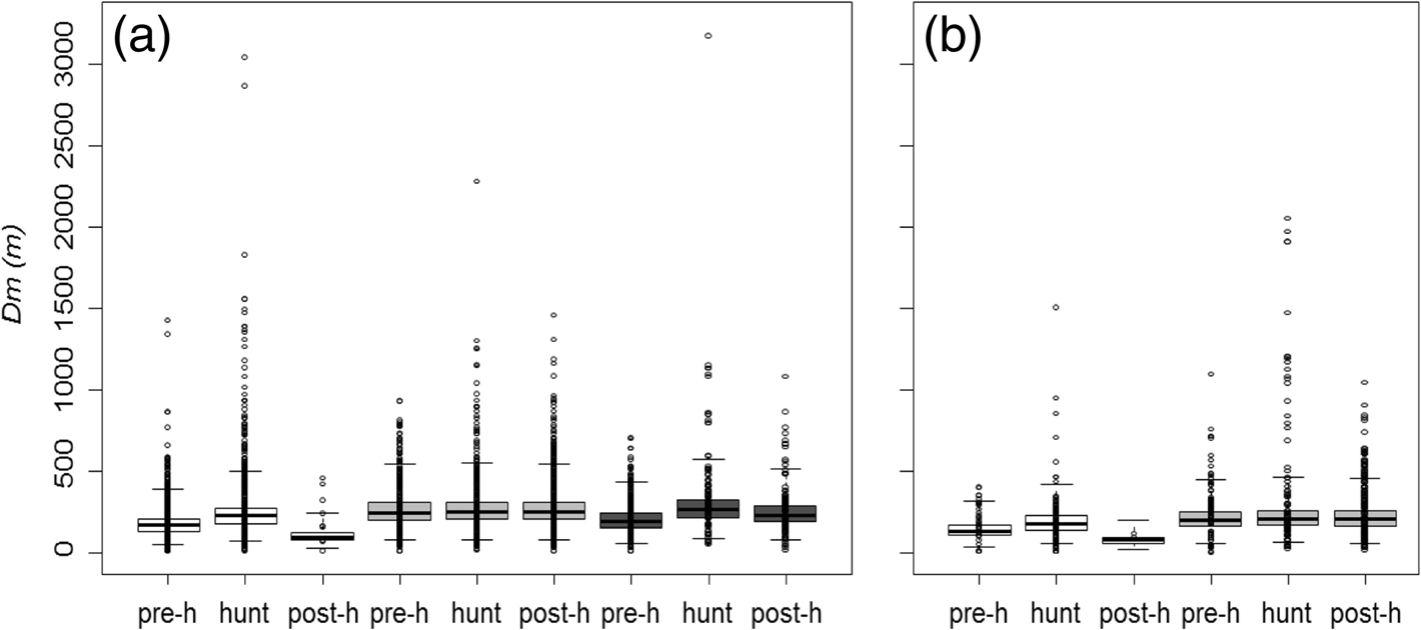
**Distances from the home-range of origin (*Dm*) centre for each period (pre-h: prehunting period; hunt: hunting period post-h: post hunting period) and each age class (white: juveniles; light grey: yearlings; dark grey: adults). a**: philopatric individuals. **b**: dispersers after settlement (no dispersers recorded as adult after settlement). The boxplots represent the distribution of the conditional mean predictions from the model Dm = age*per + disp, and the dots show the raw data.

The period effect was confirmed by modeling the distance moved (*Dm*) (Table 3). Among the set of models testing for the effects of *sex*, *age* and dispersal status, five models, all including an age effect, were retained as the most plausible (0 < ΔAICc < 2). Starting from these models and adding the factor period (hypothesis 2) always improved model fit compared to the former group of models (ΔAICc > 8 with respect to the models retained in the first step, see Additional file 4). We chose the model *Dm = age + per* as the most plausible model as model averaged predictions were close (see Additional file 5). Overall, estimates (on the transformed scale) from the model *Dm = age + per* showed that the distances from the site of origin were, within each age-class, higher during the hunting period (8.81 ± 0.18, 9.13 ± 0.19, 9.37 ± 0.42 in juveniles, yearlings and adults respectively), compared to the rest of the year (pre-hunting: 8.12 ± 0.17, 9.06 ± 0.19, 8.58 ± 0.36; post-hunting: 6.84 ± 0.56, 9.09 ± 0.17, 9.04 ± 0.41 in juveniles, yearlings and adults respectively) (Figure 5). Interestingly, yearlings and adults moved greater distances during the post-hunting period than during the pre-hunting period, whereas the reverse was true in juveniles.

**Table 3:** **Model selection for the distances moved from the home-range of origin ( *Dm *) based on AICc and Akaike weights (*w***_***i***_**)**

***Models***	***np***	***Deviance***	***AICc***	***ΔAICc***	***w***_***i***_
**Hypothesis 1**					
***Dm = age***	**6**	**10456.34**	**10469.38**	**0.00**	**0.28**
Dm = age + sex*disp	**9**	**10449.68**	**10470.01**	**0.62**	**0.20**
***Dm = age + disp***	**7**	**10454.98**	**10470.40**	**1.02**	**0.17**
***Dm = age + sex + disp***	**8**	**10452.80**	**10470.64**	**1.25**	**0.15**
***Dm = age + sex***	**7**	**10455.24**	**10470.66**	**1.28**	**0.15**
Dm = age*sex + sex*disp	11	10448.52	10474.03	4.65	0.03
Dm = age*sex	9	10454.52	10474.85	5.47	0.02
Dm = age*sex + disp	10	10452.02	10474.92	5.53	0.02
**Hypothesis 2**					
Dm = age*per	**12**	**10421.98**	**10450.20**	**0.00**	**0.21**
Dm = age*per + disp	**13**	**10419.42**	**10450.40**	**0.20**	**0.19**
Dm = age*per + sex*disp	**15**	**10414.20**	**10450.96**	**0.76**	**0.14**
Dm = age*per + sex + disp	**14**	**10417.18**	**10451.02**	**0.82**	**0.14**
Dm = age*per + disp*per	**15**	**10415.40**	**10452.15**	**1.95**	**0.08**
Dm = age*per + sex*disp + disp*per	17	10410.04	10452.91	2.71	0.05
Dm = age*per + disp*per + sex	16	10413.20	10452.96	2.76	0.05
Dm = age*per + sex*per + disp	16	10413.90	10453.67	3.47	0.04
Dm = age*per + sex*disp + disp*per	17	10411.18	10454.05	3.86	0.03

Models in bold characters are those having the best support from the data for each hypothesis. Only the the top ranked models are shown (see Additional file 4 for the complete model selection).

## Discussion

### No evidence that explorations are dispersal attempts or preludes

In the present work, we tested whether foray movements in hares could be failed dispersal attempts/dispersal preludes or, in contrast whether these movements were simply triggered by hunting related disturbance. Given that natal dispersal in hares is male biased and occurs preferentially in immature individuals (< 180 days old) [17,18], we expected under the first prediction that the propensity for making explorations would be male-biased and occur preferentially in juveniles and during the period when most natal dispersal events are recorded, i.e. from July to November [17,18]. However, we did not find strong support for a male-bias in the propensity for undertaking explorations. Instead, exploration rate was primarily explained by period and age-class, although the period effect was similar across age-classes. Explorations in juvenile hares occurred on average late in the season, regardless of the date of capture, in contrast to dispersal events. In addition, they were observed both in philopatric and dispersing juveniles after settlement. Hence, exploration movements in hares seem to share proximate factors other than natal dispersal.

An alternative hypothesis was that hunting and related disturbances could explain explorations in hares. Most explorations in juveniles were recorded soon after the beginning of hare hunting (67%, n = 24) i.e. at the end of September, while most ‘One-way’ dispersal events were recorded before the beginning of hunting, from July (70%, n = 23). The propensity for undertaking explorations in philopatric and dispersing hares after settlement was highest during the hare hunting season (October-December) but also during the following months. October-February is the roe deer hunting season. January is the beginning of the hare mating season, although the earliest hare courtships may occur in December, which coincides with the end of the hunting season [32]. During the mating season, searching for mates could trigger exploration trips. Very few individuals are fertile during the hare-hunting season [32] and thus, hare mating activity is unlikely to be the causal factor for most foray movements during the hunting period.

October-February also corresponds to harsher weather and ecological conditions. Most fields are harvested (except for some winter crops), and as result exploration forays may also occur in response to food resource limitation. However, hare home-ranges are wide [33] and encompass a broad range of field types, and thus varying food resources. In addition, if a field or a particular food resource was particularly attractive for hares, one might expect individuals to perform recurrent visits to that location. Hares have precise knowledge of their surroundings and memory of previously visited locations, as suggested by two females in our study that made recurrent back and forth trips between two well defined and distant places. Apart from these two individuals, most hares performed several explorations to different locations, and in apparent random directions, making the hypothesis of foraging trips less likely.

Although exploration rates were higher in adults than in yearlings, they moved smaller distances. Adult hares likely benefit from familiarity within their home-range compared to yearlings, philopatric and newly settled dispersers. A better knowledge of the habitat, for example where to find mates and refuges, especially during late autumn-winter periods, could explain why adults explored more but in shorter distances, buffering the cost of movements through unfamiliar habitats. Longer movements in yearlings may also reveal late dispersal preludes with unsuccessful settlement because of disturbances in the new location, even in hares that have already dispersed. For example, one individual undertook a one-way dispersal movement in the pre-hunting period, settled in a new place for few days, then dispersed again at the beginning of the hunting period to a second new home range where it settled for the remainder of the study period.

### Explorations in juvenile hares as a response to human-related predation risk?

Our results suggest that hunting and related disturbances appear to affect the use of space in European hares and trigger temporary explorations. Bray [20] also found that exploration movements were more frequent in a hunting zone than in a non-hunting zone during the hunting period. These results contrast with previous findings in this species that did not show significant temporal changes in spatial behaviour and home range size during October-December [33,34]. In both studies, home-range size estimates (MCP, Kernel) and distances of consecutive Day to Day fixes (DDD) were used to evaluate monthly or bi-monthly temporal changes in hare spacing behaviour. Herein, we combined two different measures to investigate the potential effect of hunting in spatial behaviour: (1) a discrete index of exploration based on previous home-range studies in our population [17], and (2) the consecutive distances from the centre of the home-range of origin. Both these measures allow a more subtle detection of short temporal changes in space use compared to classical home-range size estimates computed over long time periods. This may explain, in part, why the two previous studies failed to detect any temporal changes in spacing behaviour during the hunting period. Nonetheless, when considering the standard error around monthly estimates of home-range size and DDD, Reitz and Léonard [33] showed that variance was high during this time, suggesting strong heterogeneity amongst individuals. Hares faced hunting pressure in this study. In contrast, Rühe and Hohmann [34] found no increase in either home range size or DDD during this period. Hares did not face hunting pressure in their study. These results thus support our hypothesis that hunting may exert some influence on hares ‘spacing behaviour and promotes extra home-range movements in response to increasing predation risk and disturbances.

## Conclusions

In the present work, we show that exploration behaviour in hares occurs predominantly during hunting periods (hare hunting period and roe deer drive hunts), overlapping in part with the beginning of the mating season. We suggest that temporary movements outside the usual home-range may be due to a temporary increase in predation risk due to hunting, as well as searching for mating opportunities. Changes in spatial distribution and increasing movement rates due to increasing predation risk are not rare (for other examples, see [35]). Hunting is a human-related predation that may affect species behaviour and distribution in various ways [36]. Until recently, the effect of hunting has predominantly been investigated through its effect on genetics, survival and breeding parameters at the population scale [37]. Evidence is now accumulating that hunting may also have consequences at the behavioural level [38]. For instance, it has been shown that wild boars *Sus scrofa* tend to move into rescue zones during the hunting period [39]. Changes in spatial behaviour and extra home-range movements of deers have also been recorded during hunting periods [40-43]. Similar patterns seem to occur in the European hares, but detailed knowledge of the exploration process in hares is still unclear, especially due to the overlap among mating, dispersal and hunting seasons. As such, future comparisons of space use in harvested and non-harvested hare populations are warranted. In addition, if hunting is indeed the main factor triggering explorations, one might expect hares to move into rescue zones or vegetation refuges such as hedges or groves. Unfortunately, we have no precise information about the selected habitat during forays, an aspect that should be redressed in future studies. Furthermore, it is still an open question as to the role hunting and related disturbances play in individual decisions to disperse, given that around 30% of permanent dispersal events occurred during the hunting season. Also poorly understood are the factors influencing the decision to settle in a given place. These last questions are not trivial and may concern a broad range of game species, which often disperse during the hunting season (e.g. wild boar [44], and red grouse *Lagopus lagopus*[45]). This issue has rarely been specifically addressed despite the important consequences hunting may have at the population level. We strongly recommend future experimental studies to disentangle the influence of hunting from other causative factors on species dispersal. This may be particularly important in species of conservation concern. Overall, our observations and conclusions in hares emphasize the strong need to take into account human harvesting disturbances (hunting, but also crop harvesting) in future management and conservation policies of declining game species, and the realization that hunting may effect the behaviour of more than just the species targeted.

## Competing interests

The authors declare that they have no competing interests.

## Authors’ contributions

JL, YL and DP designed the hare population study; JL and YL did the field work. AA performed the statistics and wrote the manuscript. DP and JL provided comments and improvements throughout the analysis and preparation of the manuscript. All authors read and approved the final manuscript.

## Supplementary Material

Additional file 1 Trap frequency of hares according to the year. Click here for file

Additional file 2 Parameter estimates, standard errors and p-values of the linear model log(BM) = Dc*sex*disp.
Click here for file

Additional file 3 **Model averaged predictions of the top ranked models explaining variation in *dexp.***
Click here for file

Additional file 4 **Global model selection for the factors explaining variation in *Dm.***
Click here for file

Additional file 5 **Model averaged predictions of the top ranked models explaining variation in *Dm.***
Click here for file
