# Supplementary material for: Exploration forays in juvenile European hares (Lepus europaeus): dispersal preludes or hunting-induced troubles?
Source: BMC Ecol. 2014 Feb 26;14:6. doi: 10.1186/1472-6785-14-6 (PMC3943402; doi:10.1186/1472-6785-14-6)
Supplement: Additional file 1 — Trap frequency of hares according to the year. [file 1472-6785-14-6-S1.docx]

**Additional file 1**

Trap frequency of hares according to the year.
