# Supplementary material for: Exploration forays in juvenile European hares (Lepus europaeus): dispersal preludes or hunting-induced troubles?
Source: BMC Ecol. 2014 Feb 26;14:6. doi: 10.1186/1472-6785-14-6 (PMC3943402; doi:10.1186/1472-6785-14-6)
Supplement: Additional file 2 — Parameter estimates, standard errors and p-values of the linear model log(BM) = Dc*sex*disp. [file 1472-6785-14-6-S2.docx]

**Additional file 2**

Parameter estimates, standard errors (*Std error*) and their respective p-values (student t test) of the linear model *log(BM) = Dc*sex*disp.*

| ***parameter*** | ***Estimate*** | ***Std error*** | ***student t*** | ***p-value*** |
| --- | --- | --- | --- | --- |
| *Intercept* | 1.95e+03 | 3.83e+02 | 5.09 | 1.07e-05 |
| *Dc* | -9.03e-02 | 1.91 | -0.05 | 0.96 |
| *disp(O-W)* | -1.15e+03 | 1.15e+03 | -1.00 | 0.32 |
| *sexe(male)* | -1.24e+01 | 7.16e+02 | -0.02 | 0.99 |
| *Dc:disp(O-W)* | 5.29 | 5.71 | 0.93 | 0.36 |
| *Dc:sexe(male)* | 2.03 | 3.46 | 0.59 | 0.56 |
| *disp(O-W):sexe(male)* | 1.28e+03 | 1.373e+03 | 0.93 | 0.36 |
| *Dc:disp(O-W):sexe(male)* | -7.18 | 6.78 | -1.06 | 0.30 |
