# Supplementary material for: Exploration forays in juvenile European hares (Lepus europaeus): dispersal preludes or hunting-induced troubles?
Source: BMC Ecol. 2014 Feb 26;14:6. doi: 10.1186/1472-6785-14-6 (PMC3943402; doi:10.1186/1472-6785-14-6)
Supplement: Additional file 3 — Model averaged predictions of the top ranked models explaining variation in dexp. [file 1472-6785-14-6-S3.docx]

**Additional file 3**

Model averaged predictions (pred.) and respective 95 % confidence intervals (95% CI) of the top ranked models explaining variation in *dexp* according to *sex, age* (juvenile, *juv.-* yearlings, *yearl.-* adults, *ad.*), movement type and the period. Relative variable importance: *per:* 1; *age*: 0.87; *disp*: 0.37; *disp*per*:0.22; *sex*:0.17.

|  | ***Sex*** | ***female*** | | | | ***male*** | | | |
| --- | --- | --- | --- | --- | --- | --- | --- | --- | --- |
|  |  | ***dispersers*** | | ***philopatric*** | | ***dispersers*** | | ***philopatric*** | |
| ***period*** | ***age*** | pred. | *95 % CI* | pred. | *95 % CI* | pred. | *95 % CI* | pred. | *95 % CI* |
| ***pre-hunting*** | ***juv.*** | 0.06 | *0.07* | 0.06 | *0.05* | 0.07 | *0.07* | 0.06 | *0.05* |
|  | ***yearl.*** | 0.12 | *0.13* | 0.11 | *0.08* | 0.13 | *0.13* | 0.11 | *0.09* |
|  | ***ad.*** | 0.13 | *0.18* | 0.12 | *0.12* | 0.14 | *0.18* | 0.12 | *0.13* |
| ***hunting*** | ***juv.*** | 0.20 | *0.13* | 0.21 | *0.12* | 0.21 | *0.14* | 0.22 | *0.12* |
|  | ***yearl.*** | 0.34 | *0.17* | 0.35 | *0.15* | 0.35 | *0.17* | 0.36 | *0.16* |
|  | ***ad.*** | 0.35 | *0.28* | 0.37 | *0.26* | 0.37 | *0.28* | 0.38 | *0.26* |
| ***post-hunt.*** | ***juv.*** | 0.11 | *0.15* | 0.15 | *0.14* | 0.12 | *0.15* | 0.15 | *0.14* |
|  | ***yearl.*** | 0.20 | *0.18* | 0.26 | *0.15* | 0.21 | *0.19* | 0.27 | *0.15* |
|  | ***ad.*** | 0.21 | *0.25* | 0.28 | *0.23* | 0.22 | *0.26* | 0.29 | *0.23* |
