# Supplementary material for: Exploration forays in juvenile European hares (Lepus europaeus): dispersal preludes or hunting-induced troubles?
Source: BMC Ecol. 2014 Feb 26;14:6. doi: 10.1186/1472-6785-14-6 (PMC3943402; doi:10.1186/1472-6785-14-6)
Supplement: Additional file 4 — Global model selection for the factors explaining variation in Dm. [file 1472-6785-14-6-S4.docx]

**Additional file 4**

Global model selection for the factors explaining variations in *Dm*.

| ***Models Dm =*** | *np* | *Deviance* | *AICc* | *ΔAICc* | | *w_i_* |
| --- | --- | --- | --- | --- | --- | --- |
| **Hypothesis 1** |  |  |  |  |  | |
| ***age*** | **6** | **10456.34** | **10469.38** | **0.00** | | **0.28** |
| ***age + sex*disp*** | **9** | **10449.68** | **10470.01** | **0.62** | | **0.20** |
| ***age + disp*** | **7** | **10454.98** | **10470.40** | **1.02** | | **0.17** |
| ***age + sex + disp*** | **8** | **10452.80** | **10470.64** | **1.25** | | **0.15** |
| ***age + sex*** | **7** | **10455.24** | **10470.66** | **1.28** | | **0.15** |
| *age * sex + sex * disp* | 11 | 10448.52 | 10474.03 | 4.65 | | 0.03 |
| *age * sex* | 9 | 10454.52 | 10474.85 | 5.47 | | 0.02 |
| *age * sex + disp* | 10 | 10452.02 | 10474.92 | 5.53 | | 0.02 |
| *(.)* | 4 | 10490.18 | 10498.66 | 29.28 | | 0.00 |
| *sex* | 5 | 10488.50 | 10499.24 | 29.86 | | 0.00 |
| *sex * disp* | 7 | 10484.28 | 10499.70 | 30.31 | | 0.00 |
| *disp* | 5 | 10489.70 | 10500.44 | 31.06 | | 0.00 |
| *sex + disp* | 6 | 10487.44 | 10500.49 | 31.11 | | 0.00 |
| **Hypothesis 2** |  |  |  |  | |  |
| ***age*per*** | **12** | **10421.98** | **10450.20** | **0.00** | | **0.21** |
| ***age*per + disp*** | **13** | **10419.42** | **10450.40** | **0.20** | | **0.19** |
| ***age*per + sex*disp*** | **15** | **10414.20** | **10450.96** | **0.76** | | **0.14** |
| ***age*per + sex + disp*** | **14** | **10417.18** | **10451.02** | **0.82** | | **0.14** |
| ***age*per + disp*per*** | **15** | **10415.40** | **10452.15** | **1.95** | | **0.08** |
| *age*per + sex*disp + disp * per* | 17 | 10410.04 | 10452.91 | 2.71 | | 0.05 |
| *age * per + disp * per + sex* | 16 | 10413.20 | 10452.96 | 2.76 | | 0.05 |
| *age * per + sex * per + disp* | 16 | 10413.90 | 10453.67 | 3.47 | | 0.04 |
| *age * per + sex*disp + disp*per* | 17 | 10411.18 | 10454.05 | 3.86 | | 0.03 |
| *age+sex*disp + disp*per* | 13 | 10425.00 | 10456.00 | 5.80 | | 0.01 |
| *age + disp*per* | 11 | 10430.56 | 10456.07 | 5.87 | | 0.01 |
| *age + sex + disp*per* | 12 | 10428.34 | 10456.56 | 6.36 | | 0.01 |
| *age + per* | 8 | 10438.96 | 10456.80 | 6.60 | | 0.01 |
| *age + disp*sex + per* | 11 | 10431.42 | 10456.93 | 6.73 | | 0.01 |
| *age + disp + per* | 9 | 10436.82 | 10457.15 | 6.95 | | 0.01 |
| *age*per + sex * per + disp * per* | 18 | 10411.28 | 10457.34 | 7.14 | | 0.01 |
| *age + sex + disp + per* | 10 | 10434.54 | 10457.44 | 7.25 | | 0.01 |
| *age*per + sex*per + disp*sex + disp*per* | 19 | 10408.32 | 10457.66 | 7.47 | | 0.00 |
| *age + sex*per + disp*sex* | 13 | 10428.98 | 10459.96 | 9.77 | | 0.00 |
| *age +sex*per + disp* | 12 | 10431.94 | 10460.17 | 9.97 | | 0.00 |
| *age + sex*disp + sex* per + disp*per* | 15 | 10423.52 | 10460.28 | 10.08 | | 0.00 |
| *age + sex*per + disp * per* | 14 | 10426.78 | 10460.62 | 10.42 | | 0.00 |
| *age* | 6 | 10456.34 | 10469.38 | 19.19 | | 0.00 |
| *age + sex*disp* | 9 | 10449.68 | 10470.01 | 19.81 | | 0.00 |
| *age + disp* | 7 | 10454.98 | 10470.40 | 20.20 | | 0.00 |
| *age + sex + disp* | 8 | 10452.80 | 10470.64 | 20.44 | | 0.00 |
| *age + sex* | 7 | 10455.24 | 10470.66 | 20.46 | | 0.00 |
