# Supplementary material for: Exploration forays in juvenile European hares (Lepus europaeus): dispersal preludes or hunting-induced troubles?
Source: BMC Ecol. 2014 Feb 26;14:6. doi: 10.1186/1472-6785-14-6 (PMC3943402; doi:10.1186/1472-6785-14-6)
Supplement: Additional file 5 — Model averaged predictions of the top ranked models explaining variation in Dm. [file 1472-6785-14-6-S5.docx]

**Additional file 5**

Model averaged predictions (pred.) and respective 95 % confidence intervals (95% CI) of the top ranked models explaining variation in *Dm* according to *sex, age* (juvenile, *juv.-* yearlings, *yearl.-* adults, *ad.*), movement type and the period. Relative variable importance: *age:* 1; *per:* 1; *age*per:* 1, *disp:* 0.87; *sex*: 0.51; *disp*sex*: 0.32; *disp*per:* 0.17.

|  | ***Sex*** | ***female*** | | | | ***male*** | | | |
| --- | --- | --- | --- | --- | --- | --- | --- | --- | --- |
|  |  | ***dispersers*** | | ***philopatric*** | | ***dispersers*** | | ***philopatric*** | |
| ***period*** | ***age*** | pred. | *95 % CI* | pred. | *95 % CI* | pred. | *95 % CI* | pred. | *95 % CI* |
| ***pre-hunting*** | ***juv.*** | 7.86 | *0.94* | 7.67 | *0.40* | 8.14 | *0.55* | 7.58 | *0.71* |
|  | ***yearl.*** | 8.76 | *0.92* | 8.57 | *0.44* | 9.04 | *0.60* | 8.48 | *0.68* |
|  | ***ad.*** | 8.09 | *1.23* | 7.90 | *0.86* | 8.36 | *0.67* | 7.81 | *1.12* |
| ***hunting*** | ***juv.*** | 8.55 | *1.35* | 8.25 | *0.45* | 8.82 | *0.57* | 8.16 | *0.66* |
|  | ***yearl.*** | 8.85 | *0.90* | 8.55 | *0.46* | 9.13 | *0.57* | 8.46 | *0.67* |
|  | ***ad.*** | 8.85 | *1.25* | 8.54 | *0.94* | 9.12 | *0.77* | 8.45 | *1.13* |
| ***post-hunt.*** | ***juv.*** | 6.67 | *0.94* | 6.42 | *1.08* | 6.94 | *1.15* | 6.33 | *1.18* |
|  | ***yearl.*** | 8.81 | *0.95* | 8.57 | *0.43* | 9.09 | *0.57* | 8.47 | *0.63* |
|  | ***ad.*** | 8.53 | *1.27* | 8.28 | *0.93* | 8.81 | *0.76* | 8.19 | *1.12* |
